# Granulomatous Pancreas: A Case Report of Pancreatic Sarcoid

**DOI:** 10.1155/2017/1620392

**Published:** 2017-12-04

**Authors:** Tatiana Bihun, Yanet Diaz, Seth Wenig

**Affiliations:** ^1^Saba University School of Medicine, 27 Jackson Road, Suite 301, Devens, MA 01434, USA; ^2^Ross University School of Medicine, 2300 SW 145th Ave., Suite 200, Miramar, FL 33027, USA; ^3^Brookdale University Hospital and Medical Center, 1 Brookdale Plaza, Brooklyn, NY 11212, USA

## Abstract

Sarcoidosis is a chronic, systemic, noncaseating granulomatous disease process of unknown etiology. Sarcoidosis most commonly manifests in the lungs; however, gastrointestinal manifestations can occur. If in the GI tract, it is almost always found in the liver. Solitary pancreatic lesions are extremely rare, with less than 50 documented cases found in the literature. We present a case of a 61-year-old female, with a past medical history of sarcoidosis, who presented to the ER with unexpected weight loss, scleral icterus, right upper quadrant pain, and epigastric and back pain. US and MRI found a dilated common bile duct and mild dilation of the pancreatic duct, as well as a focal prominence in the head of the pancreas surrounded by areas of atrophy. A pancreaticoduodenectomy procedure was performed and fresh frozen sections were taken. The pathologist made a diagnosis of nonnecrotizing granulomatous pancreatitis. Pancreatic sarcoid is often asymptomatic and a benign finding on autopsy; however, clinicians should be mindful of pancreatic involvement when working up differential diagnosis for pancreatic masses.

## 1. Introduction

Sarcoidosis is a chronic, systemic, and noncaseating granulomatous disease of unknown origin [1, 2]. Pulmonary manifestations make up 90% of sarcoidosis diagnosis [3]. Extrapulmonary manifestations include, but are not limited to lymphoid, cardiac, cutaneous, ophthalmologic, neurologic, musculoskeletal, gastrointestinal, and renal systems [2, 4]. Sarcoidosis is predominantly seen in African Americans, affecting 1–40 per 100,000, with GI involvement more common in African Americans than Caucasians [1, 3, 4].

Granulomas found within the pancreas are rare, found in only 1–5% of patients with systemic sarcoidosis [1, 4, 5]. Isolated gastrointestinal findings are extremely uncommon, especially pancreatic cases. Lesions tend to occur in the head of the pancreas. Most cases are asymptomatic but can cause abdominal pain, jaundice, emesis, anorexia, and an increase in serum lipase levels [2, 4, 5]. Symptomatic pancreatic sarcoidosis is so rare that less than 50 cases have been reported in the literature since first described by Dr. Curran Sr. and Dr. Curran Jr. in 1950 [1].

## 2. Case Report

A 61-year-old African American Female patient with a past medical history of sarcoidosis, hypertension, and COPD presented to the Emergency Department with unexpected weight loss, scleral icterus, and a constant dull but sometimes sharp pain in the right upper epigastric quadrant and back. Lab findings showed elevated AST, ALT, pancreatic lipase, and direct bilirubin (Table 1). Patient was worked up for a differential diagnosis of pancreatitis versus cholecystitis.

**Figure 1:**
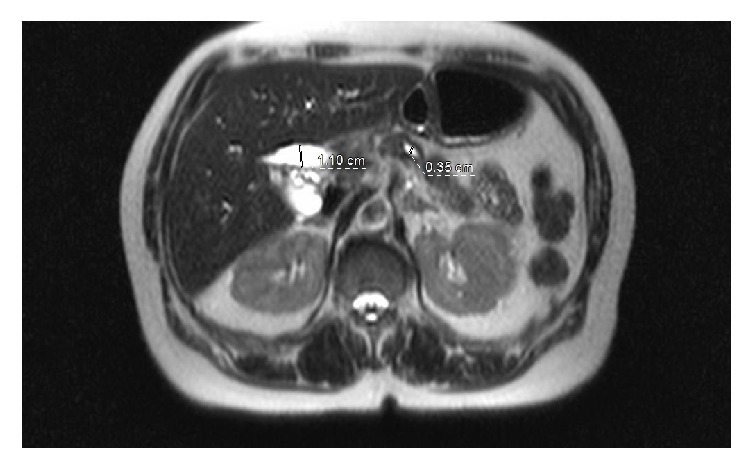
T2 axial CT: 10 mm dilated common bile duct with distended gallbladder and 3.5 mm dilated pancreatic duct.

Ultrasound of right upper quadrant found dilated common bile duct with minimal intrahepatic biliary dilation without any definite evidence of choledocholithiasis. Focal prominence was seen in the region of the pancreatic head with suggestion of atrophy within the remaining portions of visualized pancreas. MRCP was recommended. MRCP investigation found obstruction of the biliary tract as the extra hepatic CBD enters into the pancreatic head and mildly dilated pancreatic duct in body and tail. MRI confirmed this “double duct sign” finding of simultaneously dilated CBD and pancreatic duct. A normal CBD measures 4.1 mm in diameter and a normal pancreatic duct measures 3.0 mm in diameter, with the patient's CBD measuring 10 mm and pancreatic duct measuring 3.5 mm (Figure 1). This finding is suggestive of a mass in the head and neck of the pancreas.

Due to the continued symptoms of abdominal pain, the patient was scheduled for a simultaneous pancreaticoduodenectomy and needle biopsy with frozen section of mass. Patient tolerated the procedure well and no longer complained of any abdominal discomfort or pain.

**Figure 2:**
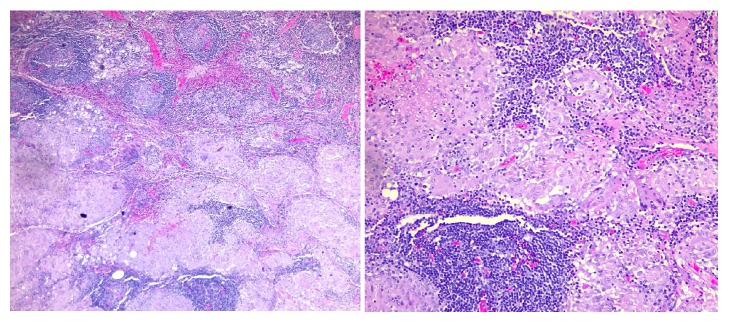
H&E stain of granulomatous lymph node at low and intermediate power.

**Figure 3:**
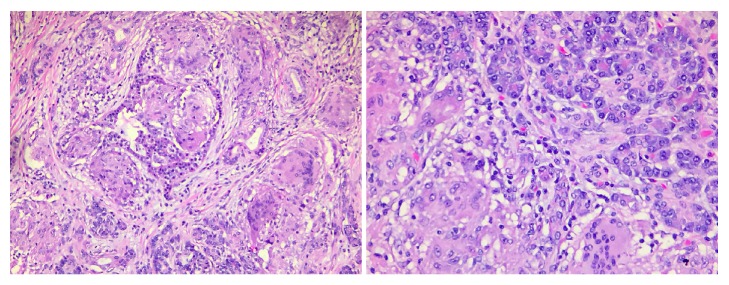
H&E stain of pancreatic mass at intermediate and high power.

On gross examination, the pancreatic mass measured 5.5 × 2.2 cm involving the bile duct and pancreatic duct. Histological samples were taken from the pancreatic tissue and lymph nodes. Lymph nodes showed reactive follicular hyperplasia, sinus histiocytosis, and scattered nonnecrotizing granulomas (Figure 2). Proximal pancreas was negative for microorganisms on AFB and GMS stains but showed nonnecrotizing granulomas compatible with patient's history of sarcoidosis (Figure 3). Gallbladder and segment of duodenum were negative for any significant pathologic change.

## 3. Discussion

Diagnosis of sarcoidosis is most commonly made on a compatible clinical-radiological picture and histological evidence of noncaseating granulomas [6]. Different diagnostic tests that can be employed to diagnose sarcoidosis include history and complete examination, CXR, PFT, certain lab values such as liver enzymes, creatinine, ACE, confirmation of granulomatous inflammation by biopsy or aspiration cytology, and microbiological examination of specimens for bacteria, acid-fast bacilli, and fungi [2, 4, 6]. There is no single test for sarcoidosis and presence of granulomas is not specific enough to establish diagnosis alone [6]. Other causes of caseating granulomas, especially if the patient is immunocompromised, must first be ruled out. Differentials include, but are not limited to, lupus erythematosus, tuberculosis, mycobacteriosis, leprosy, fungal infections, parasitic infections, foreign body reaction, drug induced granulomatosis, and histiocytosis, just to name a few [4, 6]. In this case, the patient was worked up for autoimmune diseases and evidence of immunosuppression, but all findings were either negative or within normal limits (Table 2).

Ultimately, long-term prognosis is dependent on the level of organ manifestation [6]. Corticosteroids are first-line therapy for sarcoidosis [3]. If corticosteroids are given, a tapered dose from 0.5 to 1 mg/kg can be given over the span of 1 year [1, 4]. GI treatment of granulomatous disease with corticosteroid treatment is uncertain, because corticosteroid treatment does not always reflect improvement of abnormal liver enzyme tests. One study found the out of 63 patients treated with corticosteroids, 1/3 had a complete clinical response, 1/3 had a partial response, and 1/3 showed no response at all [1]. There also has been some success with treatment using ursodeoxycholic acid or methotrexate, but data is limited [1, 3, 4]. It has been found, however, that after discontinuing corticosteroids, the recurrence rate of pancreatic sarcoidosis in severe symptomatic cases is 100% [5]. Surgical intervention is the last resort. In our patient, the severe symptomatology made her a better candidate for surgical removal and not medical therapy.

## 4. Conclusion

Sarcoidosis is a chronic, multisystemic, and noncaseating disease that rarely involves the pancreas. Pancreatic involvement is usually asymptomatic; however, symptomatic pancreatic masses do occur. To misdiagnose a pancreatic mass could be detrimental to the patient, and sarcoidosis should not be dismissed in the differential diagnosis.

## Figures and Tables

**Table 1:** Laboratory findings.

Lab findings	Presurgery	Reference range
AST	165	14–36 U/L
ALT	379	9–52 U/L
Lipase, pancreatic fluid	40	<10 U/L
Bilirubin, direct	0.6	0.0–0.4 mg/dL

**Table 2:** Immunological testing.

Lab findings	Patient	Reference rage
CA 19-9	69	<34 U/mL
CEA	<0.5	0.0–2.4 ng/mL
Smooth muscle Ab	24	<20 U
Mitochondrial Ab	Negative	Negative
ANA screen	Negative	Negative
IgG 1	820	383–929 mg/dL
IgG 2	249	241–700 mg/dL
IgG 3	64	22–178 mg/dL
IgG 4	38.3	4.0–86.0 mg/dL
Hepatitis panel	Nonreactive	Nonreactive
